# SGLT2 inhibition attenuates diabetic tubulopathy by suppressing SGK1-mediated pyroptosis

**DOI:** 10.3389/fendo.2025.1620230

**Published:** 2025-09-15

**Authors:** Xu Shi, Wei Zou, Xuehong Li, Sirui Liu, Tiantian Hu, Qiong Li, Ting Zhang, Lei Chen, Sumin Wu, Cheng Wang, Yongjie Jin

**Affiliations:** ^1^ Division of Nephrology, Department of Medicine, The Fifth Affiliated Hospital Sun Yat-Sen University, Zhuhai, China; ^2^ Guangdong Provincial Key Laboratory of Biomedical Imaging, The Fifth Affiliated Hospital Sun Yat-Sen University, Zhuhai, China; ^3^ Department of Nephrology, The University of Tokyo Hospital, The University of Tokyo, Tokyo, Japan

**Keywords:** diabetic tubulopathy, SGLT2, SGK1, pyroptosis, inflammation

## Abstract

**Background:**

Diabetic tubulopathy is increasingly recognized as a pivotal contributor to diabetic kidney disease (DKD) progression. Excessive pyroptosis of renal tubular epithelial cells exacerbates inflammation and tissue injury. Although sodium-glucose cotransporter 2 (SGLT2) inhibitors confer renal protection, their mechanistic linkage to pyroptosis remains unclear.

**Methods:**

Renal biopsies from DKD patients, STZ-induced diabetic mice, and high glucose (HG)-stimulated HK-2 cells were analyzed. Pyroptosis markers and SGK1 signaling were assessed following SGLT2 knockdown, overexpression, or treatment with SGLT2 inhibitor empagliflozin (EMPA) and the SGK1 inhibitor EMD638683 (EMD).

**Results:**

SGLT2 and Gasdermin D N-terminal domain (GSDMD-N) were upregulated in DKD kidneys and correlated with tubular injury and renal dysfunction. EMPA reduced pyroptosis marker expression, tubular injury, and fibrosis in diabetic mice. *In vitro*, HG induced SGLT2 upregulation, SGK1 activation, and pyroptosis in HK-2 cells, which were reversed by EMPA. SGLT2 overexpression increased SGK1 and pyroptosis even under normoglycemia, while SGK1 inhibition suppressed HG-induced pyroptosis and NF-κB activation.

**Conclusion:**

SGLT2 promotes diabetic tubular injury through SGK1-mediated pyroptosis. Inhibition of the SGLT2/SGK1 axis alleviates pyroptosis and offers a potential therapeutic strategy for DKD.

## 1 Introduction

Diabetic kidney disease (DKD), one of the most prevalent microvascular complications of diabetes worldwide, is a leading cause of end-stage renal disease (ESRD) (1). Traditionally, glomerular injury has been regarded as the primary site of damage in DKD, with progressive glomerular dysfunction driving proteinuria and a gradual decline in renal function (2–4). However, advancing research on DKD challenges this paradigm, suggesting that glomerular injury may not be the decisive factor in disease initiation or progression, nor the earliest event in diabetic renal injury (5, 6). Recent studies increasingly highlight the critical role of tubular pathology, particularly proximal tubular injury, in DKD progression—a phenomenon termed “diabetic tubulopathy” (7, 8).

Emerging therapeutic strategies for DKD increasingly focus on targeting tubular injury. Sodium-glucose cotransporter 2 (SGLT2) inhibitors, a unique class of antidiabetic agents primarily targeting renal tubules (9), ameliorate renal injury through glycemic control, hemodynamic modulation, metabolic regulation, sodium load reduction, and anti-inflammatory actions (10–13). Intriguingly, the anti-inflammatory benefits of SGLT2 inhibitors appear independent of their glucose-lowering effects. Randomized, double-blind, placebo-controlled, multicenter clinical trials, such as EMPA-KIDNEY, DAPA-CKD, and EMPA-HF have demonstrated that SGLT2 inhibitors exert cardiovascular and renal protective effects in both diabetic and non-diabetic populations, significantly improving patient outcomes (14–16). Transcriptomic analyses suggest that the glucose-independent renoprotection of SGLT2 inhibitors is associated with serum- and glucocorticoid-regulated kinase 1 (SGK1) (17), though further mechanistic validation is needed.

Excessive and persistent pyroptosis triggers severe inflammatory responses (18, 19). The canonical pyroptosis pathway mediated by the NOD-like receptor family pyrin domain-containing 3 (NLRP3) inflammasome plays a critical regulatory role in diseases such as DKD, obstructive nephropathy, lupus nephritis, and renal fibrosis (20, 21). Genetic ablation of NLRP3 has been demonstrated to ameliorate renal inflammation and fibrosis in diabetic mice models (22). While inflammasomes are classically associated with immune cells (e.g., mast cells, lymphocytes, and macrophages), renal tubular epithelial cells (RTECs) also express functional inflammasomes capable of secreting pro-inflammatory cytokines, positioning pyroptosis inhibition as a therapeutic strategy for diabetic tubulopathy (11, 23). RTECs act as both victims and active contributors to inflammation. Elucidating SGLT2-mediated injury mechanisms in RTECs may advance therapeutic strategies for diabetic tubulopathy.

## 2 Materials and methods

### 2.1 Human kidney biopsies and urine samples

All human samples (renal biopsies or urine) were collected from patients at the Fifth Affiliated Hospital Sun Yat-sen University after obtaining written informed consent. This study enrolled male or female patients aged ≥18 years with type 2 diabetes mellitus (T2DM), defined by the American Diabetes Association’s 2010 Standards of Medical Care in Diabetes, and biopsy-confirmed diabetic kidney disease (DKD) meeting the new pathological classification criteria provided by the Renal Pathology Society. Patients with severe non-diabetic kidney diseases, renal artery stenosis, uncontrolled hypertension, chronic heart failure with persistent symptoms, or those using SGLT2 inhibitors were excluded. Five kidney samples from tumor nephrectomy patients without diabetes or kidney diseases served as normal controls. Twenty renal biopsy samples from T2DM patients with biopsy-proven DKD were clinically characterized by persistent albuminuria (>300 mg/24 h). Demographic and clinical data of DKD patients are summarized in **Supplementary Table S1**. For urine samples, 50 mL of clean morning urine was collected from 28 biopsy-confirmed DKD patients with T2DM and 18 healthy subjects during routine physical examinations. Demographic and clinical data of DKD patients and healthy controls are presented in **Supplementary Table S2**. This study was approved by the Ethics Committee of the Fifth Affiliated Hospital Sun Yat-sen University (Approval No.: 2022#K180-1).

### 2.2 Animal models

The research involving murine models was approved by the Institutional Animal Care and Use Committee of the Fifth Affiliated Hospital Sun Yat-sen University (Approval No. 00314, Guangdong, China). Four-week-old healthy male C57BL/6J mice were purchased from the Guangdong Medical Laboratory Animal Center (Foshan, Guangdong). All mice were fed either a high-fat diet (HFD; 60 kcal% from fat, D12492, Research Diets) or a standard chow diet (12 kcal% from fat) and housed under con-trolled temperature conditions (22–26 °C) with a 12-hour light/dark cycle. After six weeks of HFD feeding, diabetes was induced in the mice by intraperitoneal injection of streptozotocin (STZ) (S0130, Sigma-Aldrich) (20). Before receiving daily STZ injections, the mice were fasted for 12 hours. STZ was administered intraperitoneally at a dose of 50 mg/kg body weight for five consecutive days. Control mice received an equal volume of 0.1 mol/L citrate buffer (pH = 4.5) simultaneously. One week after the last injection, blood samples were collected from the tail vein of all mice to measure fasting blood glucose levels. Mice with fasting blood glucose levels ≥ 300 mg/dL were considered diabetic (marked as week 0).

Mice were divided into four groups: control group, empagliflozin (EMPA) group, STZ-induced diabetes group, and STZ-induced diabetes + EMPA treatment group, with 6 mice in each group. Starting from the day fasting blood glucose testing con-firmed the successful establishment of the diabetes model (designated as week 0) until euthanasia (designated as week 12), mice were administered EMPA (HY-15409, MedChemExpress) via oral gavage at a dose of 10 mg/kg/day. Control group mice received an equal volume of 0.9% normal saline via oral gavage daily for 12 weeks.

### 2.3 Renal function

Serum creatinine levels were determined using a Creatinine Assay Kit (sarcosine oxidase) (C011-2-1, Nanjing Jiancheng Bioengineering Institute) following the manufacturer’s instructions. Similarly, Blood urea nitrogen concentrations were measured with a Urea Nitrogen Colorimetric Detection Kit (C013-2-1, Nanjing Jiancheng Bioengineering Institute) in accordance with the provided protocol.

### 2.4 Cell culture and treatment

The HK-2 cells (CRL-2190, ATCC) were cultured in DMEM/F12 medium (C11330500BT, GIBCO) supplemented with 10% FBS (C04001, Vivacell), 100 U/mL penicillin, and 100 mg/mL streptomycin (C3420-0100, Vivacell) and maintained at 37°C in a humidified incubator with 5% CO_2_. HK-2 cells were treated for 24–72 hours with either normal glucose (NG) medium (5.5 mmol/L D-glucose + 24.5 mmol/L mannitol) or high glucose (HG) medium (30 mmol/L D-glucose). SGLT2 siRNA and negative control siRNA were purchased from HanYi Bio (Guangzhou, China). The siRNA sequences used were as follows: SGLT2 sense, AGAAGGCCCUGAUU-GACAATT; SGLT2 antisense, UUGUCAAUCAGGGCCUUCUTT. The SGLT2 (SLC5A2)-3×Flag plasmid was also obtained from HanYi Bio and constructed using the pcDNA3.1 vector, with the pcDNA3.1-3×Flag plasmid serving as a negative control. siRNA and plasmid transfections were performed using Lipofectamine™ 3000 (L3000015, Thermo Fisher Scientific) according to the manufacturer’s instructions. To examine the inhibitory effects of empagliflozin (HY-15409, MedChemExpress) and EMD638683 (EMD) (HY-15193, MedChemExpress) on pyroptosis, HK-2 cells were treated with HG in the presence of EMPA (500 nM) or EMD (50 μM). After 72 hours of incubation, cells were collected for further analysis.

### 2.5 Measurement of interleukin-1β, interleukin-18, and lactate dehydrogenase

The IL-1β and IL-18 enzymatic activity in urine and cell culture medium were assayed using a IL-1β Elisa Kit (EK0392,` BOSTER Company) and IL-18 (EK0864, BOSTER Company) following the manufacturer’s instructions. The LDH release in cell culture medium was conducted by the manufacturer’s instructions.

### 2.6 Cell viability assay

The CCK-8 assay was used to assess cell viability by measuring dehydrogenase activity in live cells. HK-2 cells were seeded into 96-well plates and cultured until reached 80–90% confluency. The cells were treated with indicated agents under NG or HG conditions. To evaluate cell viability, 10 μL CCK-8 solution (CK04, Dojindo) was added to each well, followed by incubation for 2 h at 37 °C. Absorbance was measured at 450 nm wavelength using a microplate reader (EnVision).

### 2.7 Periodic acid–Schiff staining and quantitative assessment of tubular injury

PAS staining was used to assess tubular morphological changes and degree of injury. PAS staining was performed following the manufacturer’s instructions (DG0005, Leagene Biotech). Paraffin-embedded tissue sections were deparaffinized and rehydrated through sequential immersion in xylenes, graded ethanol solutions, and water, as previously described (24, 25). Oxidation was performed using 0.5% periodic acid solution for 8 minutes, followed by rinsing under running tap water for 5 minutes. Sections were then incubated with Schiff reagent for 15 minutes until a light pink coloration developed. After counterstaining with hematoxylin, the sections were dehydrated, cleared, and mounted with coverslips. Tubular injury was assessed based on morphological changes including tubular dilation or atrophy, cast formation, vacuolization, epithelial cell shedding, brush border loss, and basement membrane thickening. Tubular injury was evaluated using a semi-quantitative scoring system: 0 = no injury; 1 = ≤10% injured tubules; 2 = 11%–25%; 3 = 26%–50%; 4 = 51%–74%; and 5 = ≥75% injured (26).

### 2.8 Masson’s trichrome staining

Masson’s trichrome staining was applied to evaluate interstitial fibrosis, a hallmark of progressive kidney damage. The staining procedure was performed according to the manufacturer’s guidelines (DC0033, Leagene Biotech). Briefly, paraffin-embedded tissue sections underwent sequential deparaffinization through a graded series of xylenes and ethanol solutions followed by hydration in distilled water. Sections were incubated in Weigert’s iron hematoxylin for 5 minutes at room temperature, then thoroughly washed under running tap water. Subsequently, samples were immersed in Biebrich scarlet-acid fuchsin staining solution for 10 minutes. Differentiation was achieved by treating slides with a phosphomolybdic-phosphotungstic acid mixture for 10 minutes, followed by counter-staining with aniline blue solution. A brief 10-second acid alcohol rinse was applied to optimize cytoplasmic staining. Finally, tissues were dehydrated through an ascending ethanol series, cleared in xylene, and permanently mounted with a synthetic resin-based medium.

### 2.9 Immunohistochemistry staining

Kidney injury molecule-1 (KIM-1) is a well-recognized biomarker of tubular damage. We assessed renal injury severity through KIM-1 immunostaining. The paraffin sections were deparaffinized and rehydrated as previously described. Deparaffinized tissue sections (4μm) were prepared, and antigen retrieval was performed using citrate buffer (10 mM, C1032, Solarbio Life Sciences) in an autoclave at 120 °C for 10 min. Endogenous peroxidase activity was blocked with 1% H_2_O_2_ for 10 min. After blocking with 10% donkey serum, sections were incubated overnight at 4 °C with anti-Kim-1(R&D systems, AF1817, RRID: AB_2116446, 1:200), followed by a 1 h incubation with biotinylated secondary antibodies at room temperature and an additional 1 h incubation with HRP Conjugated Streptavidin Complex (BA1088, Boster). DAB was used for visualization, and hematoxylin was applied as a counterstain. Images were acquired using All-in-One Fluorescence Microscope (BZ-X Itasca).

### 2.10 Immunofluorescence staining

After antigen retrieval, sections were blocked with 5% donkey serum and incubated with primary antibodies overnight at 4°C: anti-SGLT2 (Abcam, sc-393350, RRID: AB_2814658, 1:200), anti-GSDMD-N (Proteintech, 66387-1-IG, RRID: AB_2881763, 1:200), anti-p-SGK1 (Affinity Biosciences, AF3001, RRID: AB_2834440, 1:200), and anti-F4/80 (CST, 70076T, RRID: AB_2799771, 1:200). Alexa Fluor 488(abcam, ab150160, RRID: AB_2756445)/594(abcam, ab150077, RRID: AB_2630356) secondary antibodies were used and nuclei were counterstained with DAPI (HY-D0814, MCE). Images were acquired on a Pannoramic 250 FLASH III scanner (3DHISTECH).

### 2.11 Immunoblotting

Immunoblotting was performed as previously described (22, 23). Briefly, the tis-sues/cells were homogenized and lysed in a lysis buffer containing protease and phosphatase inhibitors. The same amounts of protein were electrophoresed by sodium dodecyl sulfate–polyacrylamide (SDS-PAGE) gel and then transferred to PVDF mem-branes (Millipore-Sigma). The membrane blotting was performed using 5% nonfat milk for 1 h at room temperature, and then the membranes were incubated with the primary antibodies overnight at 4 °C. After washing with TBST, the membranes were incubated with HRP-conjugated goat anti-rabbit or goat anti-mouse secondary antibodies for 1 h. The signals were captured using a SuperSignal West Femto Maximum Sensitivity Substrate kit (Thermo Scientific).

The following antibodies were used in this study: anti-SGLT2 (SANTA CRUZ, sc-393350; RRID: AB_2814658, 1:1000), Cleaved Caspase-1 (CST, 4199S; RRID: AB_1903916, 1:2000), GSDMD-N (Proteintech, 66387-1-IG; RRID: AB_2881763, 1:2000), IL-1β(Abcam, ab9722; RRID: AB_308765, 1:2000), IL-18 (Abcam, ab191152, RRID: AB_2737346. 1:2000), α-tubulin(Ribobio, CRM2007, AB_2934267, 1:5000), phospho-SGK1 (Ser422) (Affinity Biosciences, AF3001, RRID: AB_2834440, 1:1000), SGK1 (CST, 12103S, RRID: AB_2687476, 1:2000), β-actin(HUABIO, HA722023, AB_3096833, 1:10,000), phospho-NF-κb(S536) (CST, 3033T, RRID: AB_331284, 1:2000), Nlrp3(CST, 15101S, RRID:AB_2722591, 1:2000), goat anti-rabbit secondary antibody (Abcam, ab205718, AB_2819160); and goat anti-mouse secondary antibody (Abcam, ab205719, RRID:AB_2755049).

### 2.12 Quantitative real-time polymerase chain reaction

Total RNA was extracted from HK-2 cells and mouse renal tissue using TRIzol reagent (15596026, Thermo Fisher Scientific). RNA purity and concentration were assessed using a NanoDrop 2000 spectrophotometer (Thermo Fisher Scientific). cDNA synthesis was carried out with the PrimeScript RT Reagent Kit (RR047A, TaKaRa Bio-technology) following the manufacturer’s protocol. Quantitative real-time PCR was performed on a CFX96 Touch Real-Time PCR Detection System (Bio-Rad) using a re-action mixture containing 12.5 μL of TB Green Premix Ex Taq II (Tli RNaseH Plus) (RR820A, TaKaRa Biotechnology), 0.4 μM of SGLT2 primers (Forward: 5’-TCCTGCTGACATCCTAGTCATT-3’, Reverse: 5’-GAAGAGCGCATTCCACTCG-3’), 2 μL of cDNA, and 8.5 μL of nuclease-free water. The thermal cycling program included an initial denaturation at 95 °C for 30 s, followed by 40 cycles of 95 °C for 5 s and 60 °C for 30 s. Gene expression levels were normalized to β-actin as an internal refer-ence and analyzed using the 2^−ΔΔCq method in Microsoft Excel.

### 2.13 Terminal deoxynucleotidyl transferase dUTP nick end labeling

Since random DNA fragmentation occurs during pyroptosis, TUNEL staining can be used to detect it (27, 28). A TUNEL BrightGreen Apoptosis Detection Kit (A112, Vazyme) was used ac-cording to the manufacturer’s instructions. Briefly, HK-2 cells were cultured on TC-treated coverslips and subjected to high-glucose (HG) treatment. After treatment, the coverslips were rinsed twice with PBS and fixed in 4% paraformaldehyde for 25 minutes at room temperature. The fixed cells were then permeabilized with 0.2% Tri-ton X-100 for 5 minutes, followed by equilibration in equilibration buffer for 30 minutes. Subsequently, the cells were incubated with 50 µL of the TdT reaction mixture at 37 °C for 60 minutes in the dark. After counterstaining with DAPI, images were acquired using All-in-One Fluorescence Microscope (BZ-X Itasca). For quantification, nine randomly selected non-overlapping fields of view were analyzed per sample. The number of TUNEL-positive cells and total cells were counted using ImageJ software, and the percentage of TUNEL-positive cells was calculated for statistical analysis.

### 2.14 Flow cytometric analysis of pyroptotic cells

Pyroptotic cells show a rapid Annexin V^+^/PI^+^ shift, due to the increased permeability of cell membranes, which allows the detection of pyroptotic cells using flow cytometry (27). HK-2 cells (2 × 10^5^ cells/well) were harvested using 0.25% trypsin without EDTA for 2 minutes. Apoptosis was assessed using the Annexin V-FITC/PI Apoptosis Detection Kit (A211, Vazyme, Nanjing, China) according to the manufacturer’s instructions. Cells were double-stained with Annexin V-FITC and propidium iodide (PI) and subsequently analyzed by flow cytometry (CytoFlex LX, Beckman, USA). For each sample, 10^5^ cells were analyzed, and each experimental condition was performed in triplicate. The percentage of pyroptotic cells was determined using FlowJo v10 software, with double-positive (Annexin V-FITC+/PI+) cells considered as pyroptotic.

### 2.15 Statistical analyses

Quantitative results are expressed as mean values ± standard error of the mean (SEM). Intergroup differences were assessed as follows: Two-group comparisons: Student’s t-test (unpaired, two-tailed) was employed. Multi-group comparisons: One-way or two-way analysis of variance (ANOVA) was selected based on experimental design, followed by Tukey’s *post hoc* test for pairwise significance evaluation when ANOVA indicated global differences (p < 0.05). A significance threshold of p < 0.05 was applied across all analyses. Data processing and statistical computations were executed using GraphPad Prism V8 software (GraphPad Inc., San Diego, CA), with normality and homogeneity of variance verified prior to parametric testing.

## 3 Results

### 3.1 SGLT2 is associated with tubular epithelial cell pyroptosis and diabetic tubulopathy

**Figure 1:**
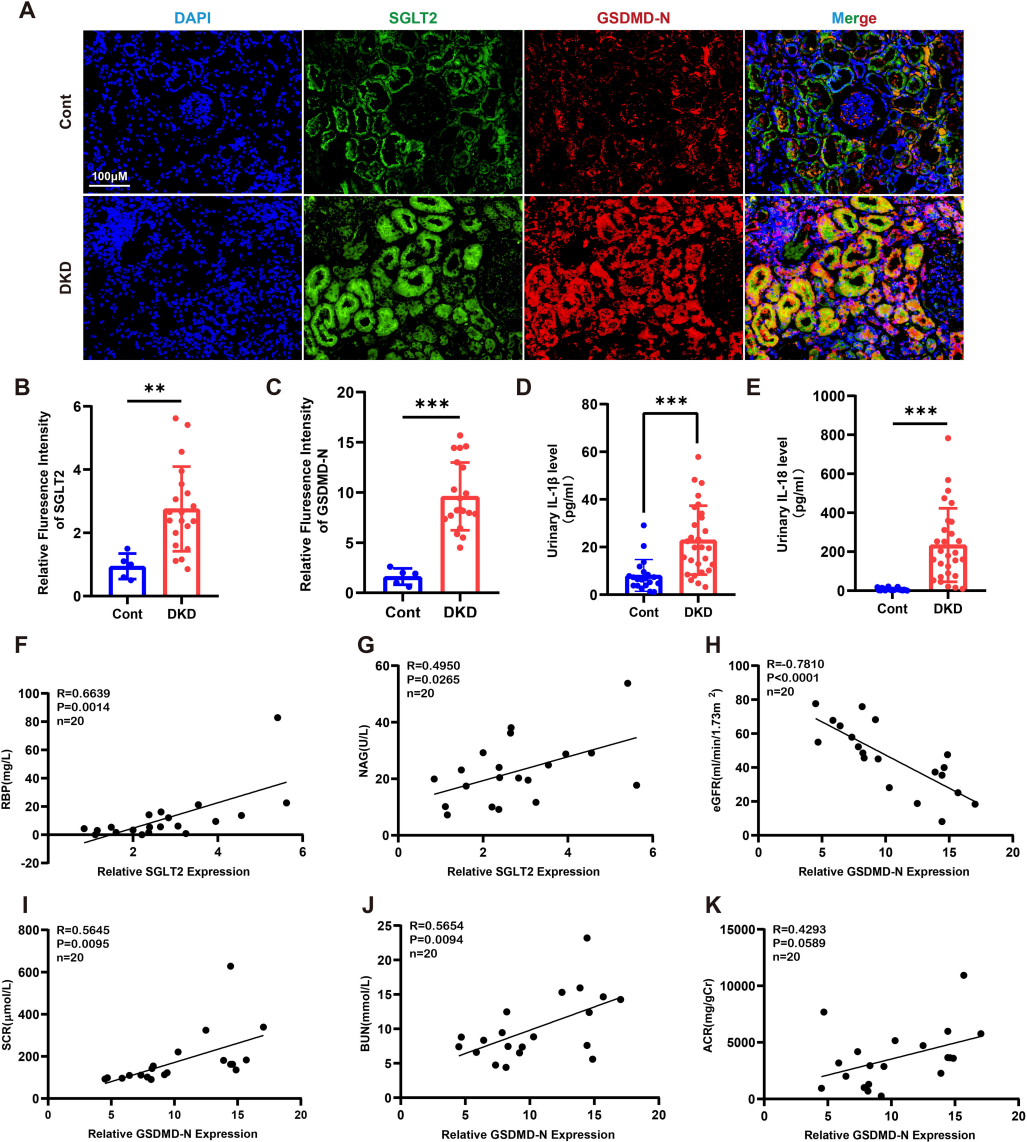
SGLT2 is associated with tubular epithelial cell pyroptosis and diabetic tubulopathy. **(A)** Representative immunofluorescence images showing co-localization of SGLT2 and GSDMD-N in kidney biopsies from DKD patients (n = 20) and paracancerous controls (n = 5). Original magnification, ×400. **(B, C)** Quantification of SGLT2 **(B)** and GSDMD-N **(C)** expression in renal tubules. **(D, E)** Urinary IL-1β **(D)** and IL-18 **(E)** levels in DKD patients (n = 28) and healthy controls (n = 20). **(F, G)** Correlation of SGLT2 expression with RBP **(F)** and NAG **(G)** in DKD patients (n = 20). **(H–K)** Correlation of GSDMD-N expression with eGFR **(H)**, SCR **(I)**, BUN **(J)**, and ACR **(K)** in DKD patients (n = 20). Data are presented as mean ± SEM. **p < 0.01, ***p < 0.001 by Student’s t test **(B–E)** or Pearson correlation analysis **(F–K)**.

To investigate the association between SGLT2 and pyroptosis, we performed dual immunofluorescence staining for SGLT2 and Gasdermin D N-terminal domain (GSDMD-N) in human renal biopsy specimens. The results demonstrated co-localization of SGLT2 and GSDMD-N in renal tubules of diabetic kidney disease (DKD) patients, with significantly increased expression compared to controls (**Figures 1A–C**). Since GSDMD-N-mediated pore formation facilitates the release of interleukin-1β (IL-1β) and IL-18—hallmark features of pyroptosis—we observed significantly elevated urinary IL-1β and IL-18 levels in DKD patients (**Figures 1D, E**). Additionally, clinical analysis revealed a positive correlation between SGLT2 expression and renal tubular injury markers, including plasma retinol-binding protein (RBP) and urinary N-acetyl-β-D-glucosaminidase (NAG) (**Figures 1F, G**). GSDMD-N expression also negatively correlated with estimated glomerular filtration rate (eGFR), and positively with serum creatinine (SCR), blood urea nitrogen (BUN), and urinary albumin-to-creatinine ratio (ACR) (**Figures 1H–K**), suggesting that SGLT2 may contribute to renal dysfunction in DKD through pyroptosis.

### 3.2 Empagliflozin attenuates tubular pyroptosis and ameliorates renal injury in STZ-induced diabetic mice

**Figure 2:**
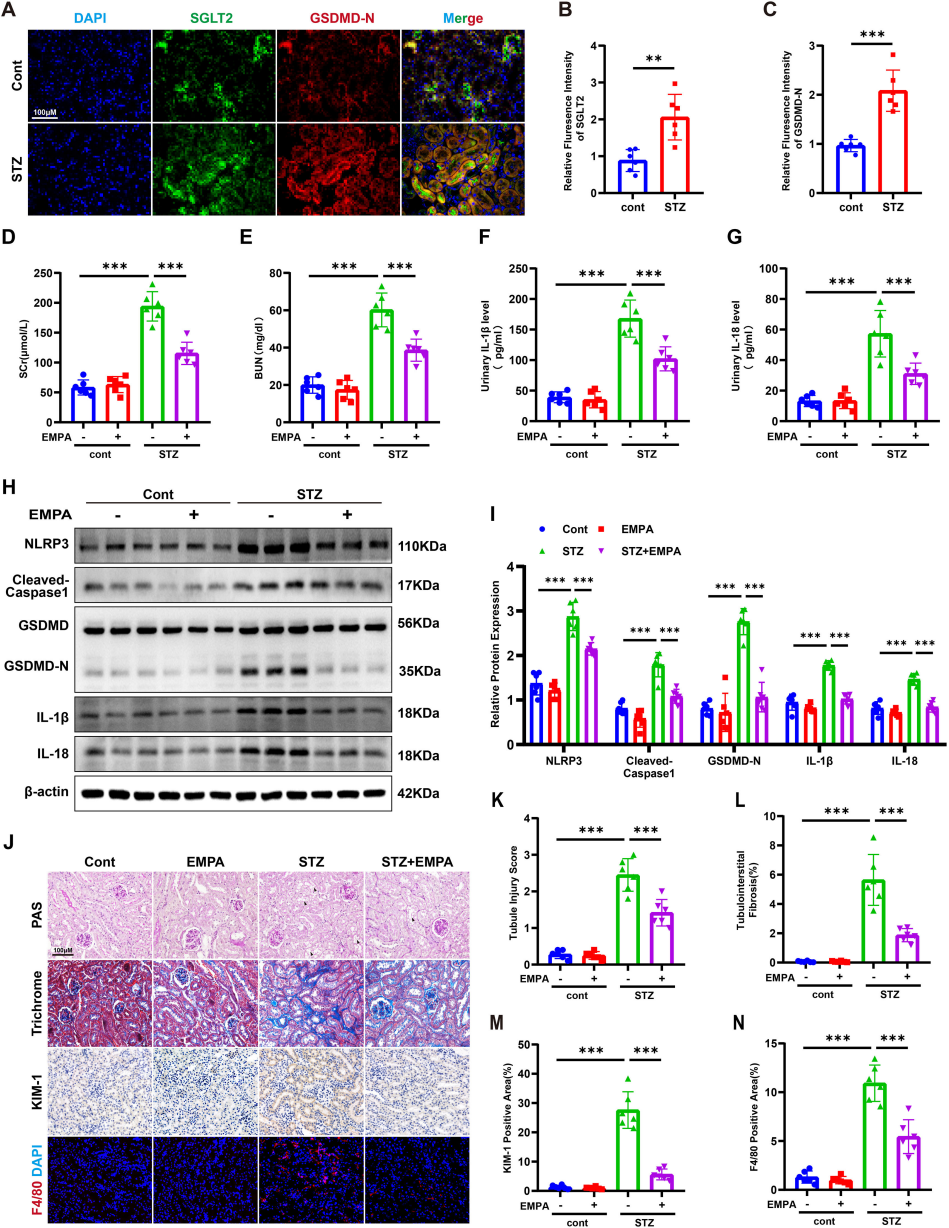
Empagliflozin attenuates tubular pyroptosis and ameliorates renal injury in STZ-induced diabetic mice. **(A)** Representative immunofluorescence images of SGLT2 and GSDMD-N in kidneys of control and diabetic mice. Original magnification, ×400. **(B, C)** Quantification of SGLT2 **(B)** and GSDMD-N **(C)** expression in renal tubules. **(D, E)** SCR **(D)** and BUN **(E)** levels. **(F, G)** Urinary IL-1β **(F)** and IL-18 **(G)** levels. **(H, I)** Western blot images **(H)** and quantification **(I)** of pyroptosis-related proteins in kidney tissue. **(J–N)** Representative images of PAS staining **(K)**, Masson’s trichrome staining **(L)**, immunohistochemistry for KIM-1 **(M)**, and F4/80 immunofluorescence **(N)**. Data are presented as mean ± SEM. **p < 0.01, ***p < 0.001 by one-way ANOVA.

In streptozotocin (STZ)-induced diabetic mice, co-localization of SGLT2 and GSDMD-N was observed in renal tubules, with significantly increased expression compared to controls (**Figures 2A–C**). STZ mice showed elevated SCR, BUN, and urinary levels of IL-1β and IL-18, all of which were mitigated by empagliflozin (EMPA) treatment (**Figures 2D–G**). Western blotting revealed marked upregulation of pyroptosis-related proteins (NLRP3, cleaved caspase-1, GSDMD-N, IL-1β, and IL-18), which was reversed by EMPA (**Figures 2H, I**). Histological staining confirmed attenuation of tubular injury and interstitial fibrosis following EMPA therapy. Immunohistochemistry showed decreased expression of KIM-1 and reduced inflammatory cell infiltration in EMPA-treated mice (**Figures 2J–N**). These findings suggest that EMPA mitigates diabetic tubulopathy by suppressing tubular pyroptosis.

### 3.3 High glucose induces SGLT2 upregulation and pyroptosis in HK-2 cells

**Figure 3:**
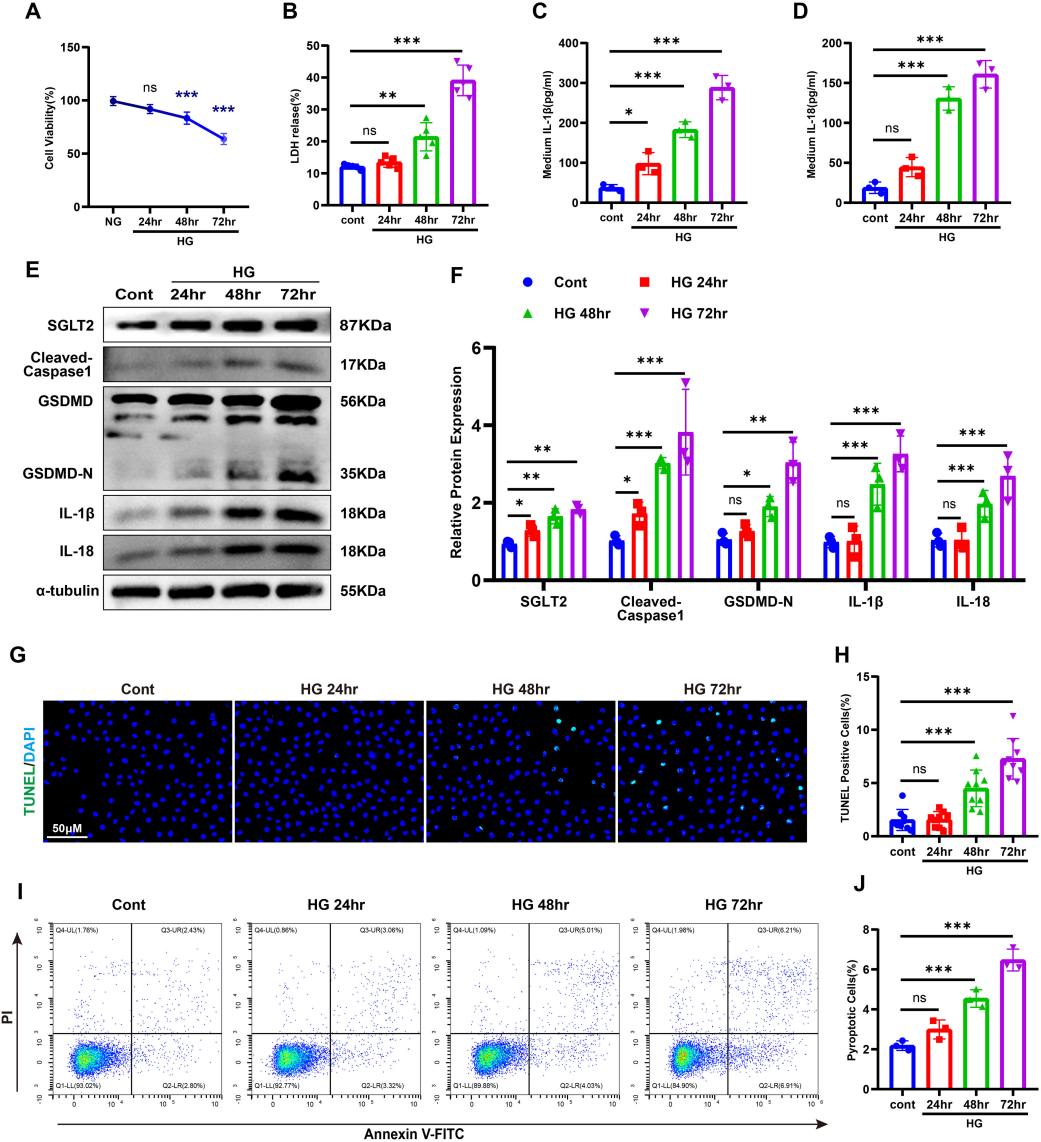
HG–induced pyroptosis in HK-2 cells. **(A)** Cell viability under HG stimulation. **(B–D)** LDH **(B)**, IL-1β **(C)**, and IL-18 **(D)** release after HG treatment. **(E, F)** Western blot images **(E)** and quantification **(F)** of SGLT2 and pyroptosis-related proteins. **(G, H)** TUNEL staining images **(G)** and quantification of TUNEL-positive cells **(H)**. **(I, J)** Annexin V-FITC/PI staining images **(I)** and quantification of pyroptotic cells **(J)**. Data are presented as mean ± SEM. *p < 0.05, **p < 0.01, ***p < 0.001, ns = not significant by one-way ANOVA.

To further validate the association between SGLT2 and pyroptosis in renal tubular epithelial cells (RTECs), HK-2 cells were exposed to high glucose (HG, 30 mM) for 24–72 hours. Cell viability declined significantly after 48 hours and dropped to ~60% by 72 hours (**Figure 3A**). Lactate dehydrogenase (LDH), IL-1β and IL-18 levels in the supernatant increased in a time-dependent manner (**Figures 3B–D**). Western blotting showed upregulation of SGLT2 and pyroptosis-related proteins, peaking at 72 hours (**Figures 3E, F**). DNA damage and membrane integrity disruption were confirmed via TUNEL and Annexin V-FITC/PI staining (27) (**Figures 3G–J**), establishing that HG induces pyroptosis in HK-2 cells, with 72-hour exposure as the optimal model condition.

### 3.4 Empagliflozin suppresses high glucose-induced pyroptosis

**Figure 4:**
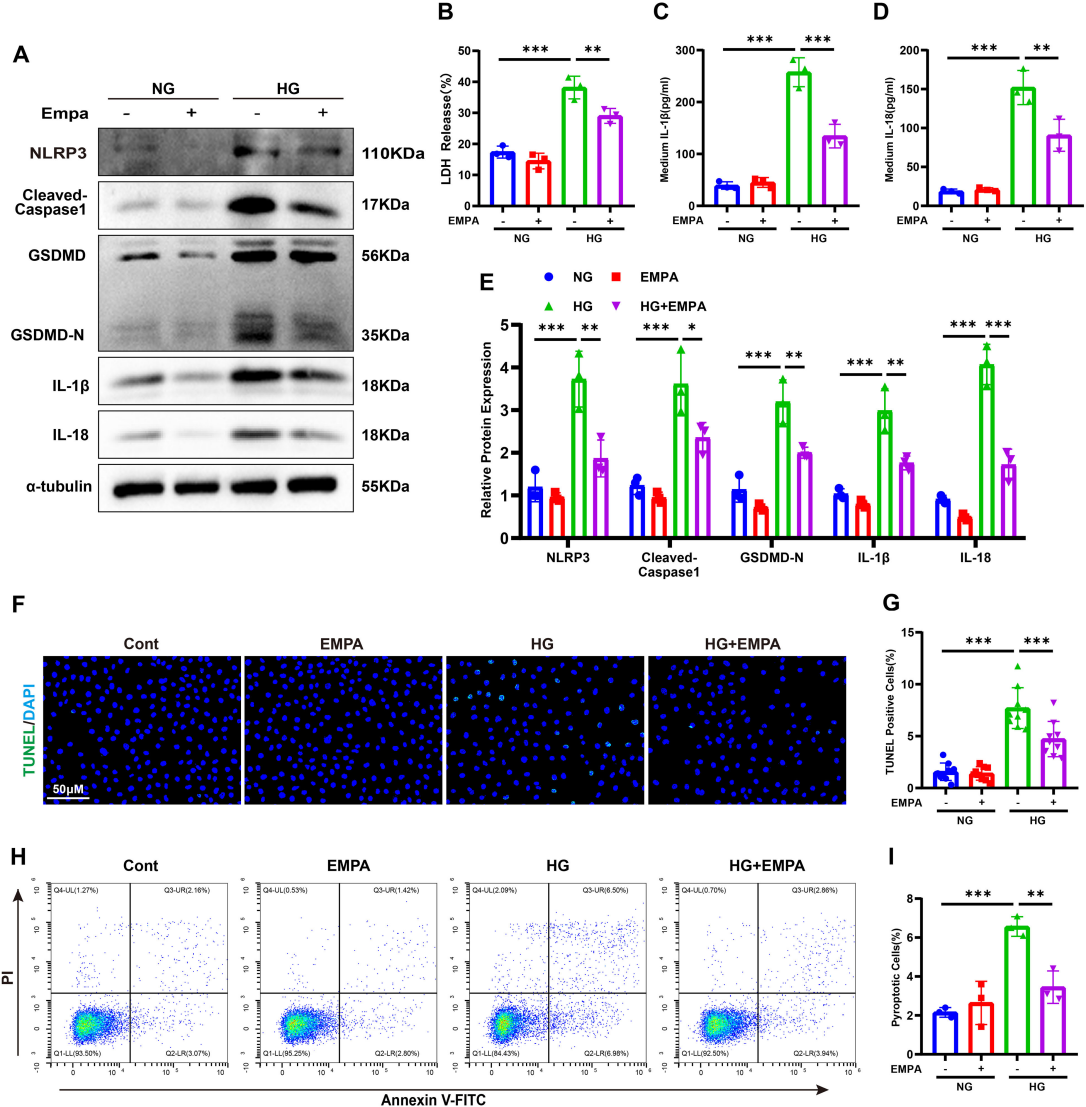
Empagliflozin suppresses HG-induced pyroptosis in HK-2 cells. **(A, E)** Western blot images **(A)** and quantification **(E)** of pyroptosis-related proteins. **(B–D)** LDH **(B)**, IL-1β **(C)**, and IL-18 **(D)** release under HG ± EMPA conditions. **(F, G)** TUNEL staining images **(F)** and quantification of TUNEL-positive cells **(G)**. **(H, I)** Annexin V-FITC/PI staining images **(H)** and quantification of pyroptotic cells **(I)**. Data are presented as mean ± SEM. *p < 0.05, **p < 0.01, ***p < 0.001 by one-way ANOVA.

To evaluate the anti-pyroptotic effects of SGLT2 inhibition, HK-2 cells under HG or NG conditions were treated with EMPA. EMPA significantly suppressed the HG-induced upregulation of NLRP3, cleaved caspase-1, GSDMD-N, IL-1β, and IL-18 (**Figures 4A, E**). It also reduced IL-1β, IL-18, and LDH release (**Figures 4B–D**). Moreover, EMPA treatment led to fewer TUNEL- and Annexin V-FITC/PI-positive cells under HG conditions (**Figures 4F–I**), confirming its *in vitro* anti-pyroptotic efficacy.

### 3.5 SGLT2 regulates SGK1 signaling and pyroptosis

**Figure 5:**
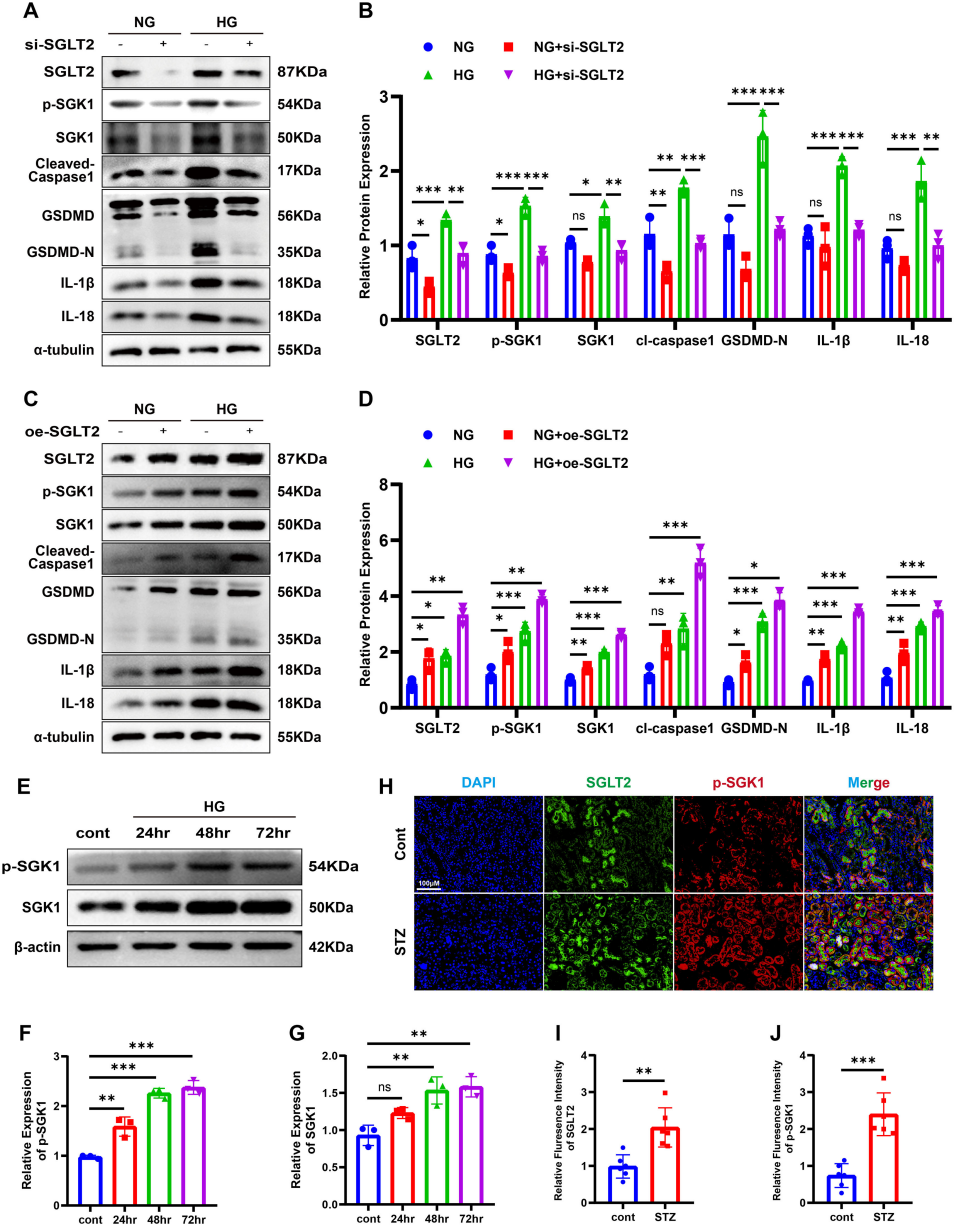
SGLT2 regulates SGK1 signaling and pyroptosis. **(A, B)** Western blot images **(A)** and quantification **(B)** of pyroptosis-related proteins in SGLT2 knockdown cells. **(C, D)** Western blot images **(C)** and quantification **(D)** in SGLT2-overexpressing cells. **(E–G)** Western blot images **(E)** and quantification of p-SGK1 **(F)** and total SGK1 **(G)** in a time-dependent manner following HG stimulation. **(H–J)** Representative immunofluorescence images showing co-localization of SGLT2 and p-SGK1 in kidneys **(H)** and their quantification **(I, J)**. Original magnification, ×400. Data are presented as mean ± SEM. *p < 0.05, **p < 0.01, ***p < 0.001, ns = not significant by one-way ANOVA or t test.

To explore whether SGLT2 modulates pyroptosis, we performed knockdown and overexpression experiments (see **Supplementary Figure S1**). SGLT2 knockdown attenuated HG-induced expression of cleaved caspase-1, GSDMD-N, IL-1β, and IL-18, while overexpression increased these markers (**Figures 5A–D**). Given evidence implicating SGK1 in SGLT2i-mediated anti-inflammatory effects, we assessed SGK1 activation (17). In HG-treated HK-2 cells, SGK1 and phosphorylated SGK1 (p-SGK1) increased in a time-dependent manner (**Figures 5E–G**), while EMPA treatment decreased both (see **Supplementary Figure S2**). Co-localization of SGLT2 and p-SGK1 was also observed in renal tubules of STZ-induced mice (**Figures 5H–J**). Notably, SGLT2 overexpression upregulated SGK1 phosphorylation even in normoglycemic conditions, suggesting SGLT2 directly regulates SGK1 signaling to promote pyroptosis.

### 3.6 SGK1 inhibition attenuates pyroptosis

**Figure 6:**
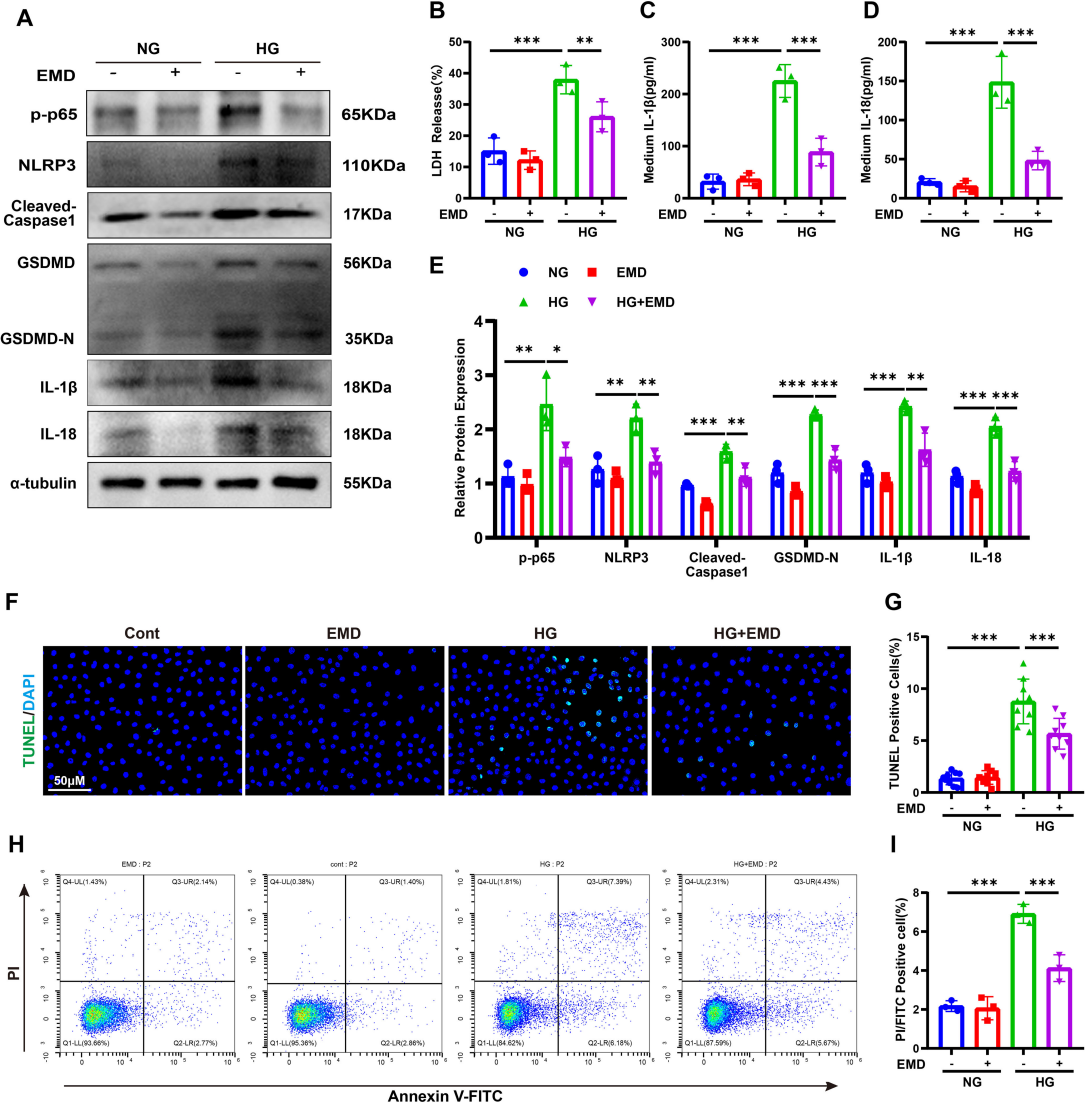
SGK1 inhibition attenuates pyroptosis in HK-2 cells. **(A, E)** Western blot images **(A)** and quantification **(E)** of pyroptosis-related proteins following SGK1 inhibition (EMD). **(B–D)** LDH **(B)**, IL-1β **(C)**, and IL-18 **(D)** release under HG ± EMD conditions. **(F, G)** TUNEL staining images **(F)** and quantification of TUNEL-positive cells **(G)**. **(H, I)** Annexin V-FITC/PI staining images **(H)** and quantification of pyroptotic cells **(I)**. Data are presented as mean ± SEM. *p < 0.05, **p < 0.01, ***p < 0.001 by one-way ANOVA.

SGK1 is known to regulate nuclear factor kappa-light-chain-enhancer of activated B cells (NF-κB), particularly its p65 (RelA) subunit, which drives NLRP3 inflammasome expression (29). To assess the role of SGK1 in tubular pyroptosis, we treated HG-stimulated HK-2 cells with EMD638683 (EMD), a selective SGK1 inhibitor. EMD reversed HG-induced upregulation of p-p65, NLRP3, cleaved caspase-1, GSDMD-N, IL-1β, and IL-18 (**Figures 6A, E**). LDH release and secretion of IL-1β/IL-18 were also reduced (**Figures 6B–D**). Additionally, EMD significantly decreased the proportion of TUNEL- and Annexin V-FITC/PI-positive cells (**Figures 6F–I**). These results demonstrate that SGK1 is a critical mediator of SGLT2-induced pyroptosis.

## 4 Discussion

Here, we demonstrate that SGLT2 promotes pyroptosis in RTECs through activation of the SGK1 pathway, contributing to diabetic tubulopathy. In DKD patients and STZ-induced mice, SGLT2 co-localized with GSDMD-N in renal tubules, accompanied by elevated urinary IL-1β and IL-18 and impaired renal function. HG induced SGLT2 and pyroptosis-related proteins in HK-2 cells, while EMPA or SGLT2 knockdown suppressed these effects. EMPA also reduced tubular injury and inflammation in diabetic mice. Mechanistically, SGLT2 enhanced SGK1 phosphorylation and pyroptosis, even under normoglycemia, whereas SGK1 inhibition by EMD reversed HG-induced pyroptosis. These findings reveal a novel SGLT2/SGK1–NLRP3 axis driving tubular injury and highlight its therapeutic relevance.

DKD has traditionally been attributed to glomerular injury, with progressive proteinuria and renal dysfunction considered hallmarks of disease progression (2–4). However, this paradigm fails to explain clinical observations, including the absence of proteinuria in 20.5%–61% of diabetic patients prior to renal function decline, and the stronger correlation of renal insufficiency with tubular pathology (e.g., tubular atrophy and interstitial fibrosis) rather than glomerular changes (5, 6, 30). Under diabetic conditions, RTECs are exposed to high glucose concentrations from both the apical and basolateral sides but lack mechanisms to limit glucose uptake, resulting in intracellular glucose overload. This burden is mostly driven by upregulated SGLT2, which enhances glucose and sodium reabsorption and promotes oxidative stress and inflammation. Based on these features, SGLT2 is thought to be closely linked to the development of diabetic tubulopathy (10, 31, 32).

Previous studies have demonstrated that empagliflozin attenuates cardiac dysfunction by reducing NLRP3 inflammasome activation in heart failure models, and that SGLT2 inhibitors counteract NLRP3 activation via the immunomodulatory metabolite itaconate in ischemia-reperfusion injury (IRI) models. While these findings support the anti-inflammatory effects of SGLT2 inhibitors in various organs, research specifically linking SGLT2 activity to pyroptosis in diabetic renal tubular epithelial cells remains limited (33, 34). Notably, pyroptosis has emerged as a critical driver of DKD progression, largely due to its uniquely inflammatory nature. Unlike other forms of regulated cell death, pyroptosis leads to cell membrane rupture and the release of pro-inflammatory cytokines such as IL-1β and IL-18, thereby amplifying tubular inflammation and injury (18–21). Mechanistically, this process is initiated by the activation of NLRP3 inflammasomes in response to damage- or pathogen-associated molecular patterns (DAMPs or PAMPs), leading to caspase-1 activation. Caspase-1 subsequently cleaves GSDMD, producing the pore-forming N-terminal fragment (GSDMD-N) that facilitates cytokine release (18). Accumulating preclinical and clinical evidence reveals that SGLT2 inhibitors exert systemic and tissue-specific anti-inflammatory effects by suppressing NLRP3 inflammasome activation (33–36). In this study, we observed co-localization of SGLT2 and GSDMD-N in renal tubules of both human DKD specimens and STZ-induced diabetic mice, along with elevated urinary IL-1β and IL-18. SGLT2 inhibition with empagliflozin reduced pyroptosis and improved renal function in diabetic mice. These findings support the hypothesis that pyroptosis, likely driven by SGLT2-mediated NLRP3 activation, contributes to diabetic tubulopathy and that SGLT2 inhibition may confer renoprotection by suppressing this process.

SGLT2 inhibitors have emerged as novel therapeutic agents for DKD. Findings from the clinical trials demonstrated that SGLT2 inhibitors improve both cardiovascular and renal outcomes not only in diabetic patients but also in those without diabetes (14–16). These observations imply that the renoprotective effects of SGLT2 inhibition are not solely dependent on glucose control. It has been proposed that part of these benefits may be attributed to anti-inflammatory activity at the kidney level (37, 38); however, the underlying molecular mechanisms remain incompletely understood. Pirklbauer et al. (17) revealed through transcriptomic analyses that SGK1 may mediate glucose-independent anti-inflammatory mechanisms of SGLT2i. SGK1, a ubiquitously expressed serine/threonine kinase of the AGC family, is crucial for glucose homeostasis. Notably, SGK1 expression is characterized by remarkably high transcriptional volatility and is regulated by a variety of physiological and pathological stimuli, including hyperglycemia, cell shrinkage, ischemia, glucocorticoids, and mineralocorticoids (39, 40). SGK1 activation occurs downstream of insulin and various growth factors, primarily via the phosphatidylinositol 3-kinase (PI3K) pathway, involving 3-phosphoinositide-dependent kinase-1 (PDK1) and mammalian target of rapamycin (mTOR). These features underscore SGK1 as a key metabolic and stress-responsive kinase, linking upstream signals such as hyperglycemia to downstream cellular processes including inflammation and cell death (39, 41). Mechanistically, SGK1 enhances NF-κB activity via phosphorylation of IKKα, and NF-κB—a master transcriptional regulator of inflammation—directly promotes the expression of NLRP3, GSDMD, IL-1β, and IL-18 (29, 42). In our study, we confirmed the co-localization of SGLT2 and SGK1 in the kidneys of STZ-induced diabetic mice. By knocking down or overexpressing SGLT2, we demonstrated that SGK1 is regulated by SGLT2. Consistently, overexpression of SGLT2 led to increased expression of SGK1 and pyroptosis markers even in the absence of high glucose stimulation. Our findings are in line with previous studies demonstrating the functional relevance of the SGLT2–SGK1 axis in diabetic kidney disease. For instance, SGLT2 knockdown has been shown to restore the Th17/Treg balance and attenuate diabetic nephropathy in *db/db* mice by regulating SGK1 via sodium signaling (43). These findings suggest that SGLT2 can directly induce pyroptosis in renal tubular epithelial cells via SGK1 activation, which may partially explain the renoprotective effects of SGLT2 inhibitors observed in non-diabetic patients. In addition, SGK1-mediated suppression of AMP-activated protein kinase activity may also be involved in the anti-inflammatory mechanisms of SGLT2 inhibitors, although this hypothesis requires further investigation (12, 13, 44).

Taken together, our study elucidates the critical role of SGLT2 in regulating pyroptosis in renal tubular epithelial cells and links it to the pathogenesis of diabetic tubulopathy. We demonstrate that SGLT2 inhibitors primarily suppress pyroptosis by modulating the SGK1 signaling pathway, and this protective effect appears to be independent of glucose levels. These findings not only deepen our understanding of the mechanisms underlying diabetic tubulopathy but also underscore the potential value of SGLT2 inhibitors as anti-inflammatory agents for treating renal tubular injury.

## Data Availability

The datasets presented in this study can be found in online repositories. The names of the repository/repositories and accession number(s) can be found below: DOI: 10.5281/zenodo.15163324.
