# Supplementary material for: SGLT2 inhibition attenuates diabetic tubulopathy by suppressing SGK1-mediated pyroptosis
Source: Front Endocrinol (Lausanne). 2025 Sep 15;16:1620230. doi: 10.3389/fendo.2025.1620230 (PMC12476994; doi:10.3389/fendo.2025.1620230)
Supplement: Supplementary file 1 [file DataSheet1.docx]

Supplementary Material

# Supplementary Tables

**1.1 Supplementary table 1:** Parameter values of DKD patients

| **No.** | **Sex** | **Age** | **eGFR**  **ml/min** | **BUN**  **mmol/L** | **SCr**  **μmol/L** | **ACR**  **mg/gCr** | **HbA1c**  **%** | **β2-MG**  **mg/L** | **α1-MG**  **mg/L** | **RBP**  **mg/L** | **NAG**  **U/L** |
| --- | --- | --- | --- | --- | --- | --- | --- | --- | --- | --- | --- |
| 1 | M | 67 | 75.87 | 4.4 | 90 | 693.31 | 6 | 0.03 | 8.35 | 0.03 | 10.03 |
| 2 | M | 47 | 45.61 | 7.45 | 154 | 2942.82 | 7.5 | 16.62 | 61.54 | 12.07 | 20.26 |
| 3 | M | 45 | 18.82 | 15.3 | 324 | 4721.37 | 7.1 | 16.7 | 89.73 | 21.29 | 24.85 |
| 4 | M | 57 | 39.99 | 12.39 | 162 | 3645.15 | 7.8 | 4.95 | 47.62 | 9.42 | 28.79 |
| 5 | M | 50 | 8.16 | 23.19 | 628 | 3651.02 | 6 | 12.21 | 72.29 | 22.5 | 17.72 |
| 6 | F | 58 | 54.96 | 8.8 | 98 | 7681.1 | 6.3 | 12.61 | 49.67 | 16.1 | 38.07 |
| 7 | M | 48 | 37.26 | 15.94 | 181 | 2271.88 | 10.5 | 6.81 | 29.87 | 5.33 | 20.42 |
| 8 | M | 40 | 77.61 | 7.4 | 92 | 943.45 | 9.2 | 0.63 | 26.79 | 4.31 | 19.92 |
| 9 | M | 43 | 68.2 | 6.5 | 113 | 257.91 | 7 | 0.01 | 5.7 | 0.03 | 10.15 |
| 10 | M | 72 | 67.75 | 6.6 | 96 | 3176.9 | 8.4 | 1.8 | 15.02 | 0.88 | 11.66 |
| 11 | F | 34 | 35.43 | 7.6 | 162 | 5985.5 | 7.4 | 5.98 | 40.23 | 5.62 | 36.18 |
| 12 | M | 52 | 48.57 | 12.46 | 142 | 1295.44 | 7.8 | 0.15 | 17.43 | 1.6 | 17.43 |
| 13 | F | 59 | 52.24 | 9.44 | 101.6 | 1010.9 | 8.3 | 5.24 | 20.5 | 1.5 | 9.2 |
| 14 | M | 63 | 47.54 | 5.6 | 135.6 | 3588.54 | 5.7 | 33.3 | 106 | 14.1 | 24 |
| 15 | F | 48 | 45.01 | 7.36 | 122.5 | 2869.16 | 5.8 | 2.66 | 16.5 | 3.4 | 29.2 |
| 16 | M | 40 | 18.34 | 14.25 | 338.8 | 5771.02 | 13.7 | 11,7 | 65 | 13.6 | 29.1 |
| 17 | F | 62 | 25.15 | 14.65 | 182.8 | 10927.5 | 5.2 | 45.9 | 165 | 82.8 | 53.8 |
| 18 | M | 54 | 28.13 | 8.85 | 220.5 | 5158.4 | 6.6 | 9.31 | 32.8 | 6.1 | 19.5 |
| 19 | M | 56 | 64.52 | 8.35 | 109.7 | 2009.21 | 8 | 3.12 | 22.8 | 3 | 7.2 |
| 20 | M | 70 | 57.9 | 4.74 | 110.6 | 4185.18 | 10.6 | 3.33 | 46.5 | 5.4 | 23.1 |

**eGFR:** Estimated Glomerular Filtration Rate, **BUN:** Blood Urea Nitrogen, **SCr:** Serum Creatinine, **ACR:** Albumin-to-Creatinine Ratio, **HbA1c:** Hemoglobin A1c, **β2-MG:** Beta-2 Microglobulin, **α1-MG:** Alpha-1 Microglobulin, **RBP:** Retinol-Binding Protein, **NAG:** N-Acetyl-Beta-D-Glucosaminidase.

**1.2 Supplementary table 2:** Parameter values of healthy subjects and DKD patients

Parameter values of healthy subjects

| **No.** | **Sex** | **Age** | **Urinary IL-1β**  **(pg/ml)** | **Urinary IL-18**  **(pg/ml)** |
| --- | --- | --- | --- | --- |
| 1 | 25 | M | 3.9 | 1.28 |
| 2 | 34 | F | 11.81 | 1.43 |
| 3 | 40 | M | 2.63 | 4.88 |
| 4 | 29 | M | 1.16 | 3.32 |
| 5 | 55 | F | 3.81 | 7.54 |
| 6 | 22 | F | 6.33 | 2.32 |
| 7 | 38 | M | 4.78 | 0.69 |
| 8 | 47 | M | 3.74 | 19.29 |
| 9 | 19 | M | 6.83 | 3.38 |
| 10 | 31 | F | 7.83 | 9.21 |
| 11 | 60 | F | 1.29 | 1.28 |
| 12 | 26 | M | 8.38 | 3.39 |
| 13 | 42 | M | 6.48 | 5.48 |
| 14 | 36 | F | 7.37 | 5.58 |
| 15 | 48 | M | 7.33 | 18.02 |
| 16 | 53 | M | 29.12 | 2.47 |
| 17 | 24 | F | 9.58 | 14.68 |
| 18 | 66 | M | 13.67 | 7.91 |

Continues on next page.

Parameter values of DKD subjects

| **No.** | **Sex** | **Age** | **Urinary**  **IL-1β**  **(pg/ml)** | **Urinary**  **IL-18**  **(pg/ml)** | **HbA1c**  **(%)** | **eGFR**  **(ml/min)** | **BUN**  **(mmol/L)** | **SCr**  **(umol/L)** |
| --- | --- | --- | --- | --- | --- | --- | --- | --- |
| 1 | M | 50 | 23.97 | 9.43 | 6 | 8.16 | 23.19 | 628 |
| 2 | M | 67 | 19.84 | 194.79 | 6 | 75.87 | 4.4 | 90 |
| 3 | M | 47 | 12.64 | 782.99 | 7.5 | 45.61 | 7.45 | 154 |
| 4 | M | 45 | 10.15 | 89.23 | 7.1 | 18.82 | 15.3 | 324 |
| 5 | M | 57 | 7.99 | 310.99 | 6 | 36.43 | 10.1 | 175 |
| 6 | M | 57 | 8.46 | 20.99 | 7.8 | 39.99 | 12.39 | 162 |
| 7 | F | 58 | 9.37 | 75.23 | 6.3 | 54.96 | 8.8 | 98 |
| 8 | M | 69 | 6.13 | 124.99 | 7.1 | 36.22 | 8.31 | 164 |
| 9 | M | 64 | 3.25 | 254.35 | 7 | 65.06 | 3.48 | 104 |
| 10 | M | 48 | 4.79 | 203.99 | 10.5 | 37.26 | 15.94 | 181 |
| 11 | F | 48 | 26.73 | 160.46 | 6.5 | 109.54 | 3.8 | 51 |
| 12 | M | 54 | 12.91 | 15.35 | 8.9 | 28.06 | 14.1 | 221 |
| 13 | F | 68 | 23.26 | 45.68 | 7.5 | 89.1 | 5 | 62 |
| 14 | M | 40 | 15.20 | 359.23 | 9.2 | 77.61 | 7.4 | 92 |
| 15 | M | 37 | 36.75 | 138.46 | 5.8 | 49.32 | 8.12 | 153 |
| 16 | M | 43 | 37.88 | 155.12 | 7 | 68.2 | 6.5 | 113 |
| 17 | M | 54 | 41.58 | 253.12 | 6.6 | 58.12 | 7.3 | 121 |
| 18 | M | 72 | 14.45 | 70.46 | 8.4 | 67.75 | 6.6 | 96 |
| 19 | F | 34 | 57.84 | 292.12 | 7.4 | 35.43 | 7.6 | 162 |
| 20 | F | 50 | 29.21 | 513.32 | 8 | 13.3 | 13.2 | 332 |
| 21 | M | 53 | 32.35 | 179.88 | 6.5 | 100.67 | 5.14 | 73 |
| 22 | M | 52 | 22.46 | 252.23 | 7.8 | 48.57 | 12.46 | 142 |
| 23 | F | 59 | 19.48 | 53.32 | 8.3 | 52.24 | 9.44 | 101.6 |
| 24 | M | 59 | 46.91 | 159.46 | 7.8 | 26.82 | 9 | 222.8 |
| 25 | M | 54 | 48.22 | 474.65 | 6.6 | 28.13 | 8.85 | 220.5 |
| 26 | M | 44 | 34.13 | 567.54 | 5.6 | 24.54 | 14.28 | 261.7 |
| 27 | M | 63 | 19.87 | 353.68 | 5.7 | 47.54 | 5.6 | 135.6 |
| 28 | M | 67 | 15.69 | 450.79 | 6.3 | 31.85 | 7.68 | 184.5 |

**eGFR:** Estimated Glomerular Filtration Rate, **BUN:** Blood Urea Nitrogen, **SCr:** Serum Creatinine, **ACR:** Albumin-to-Creatinine Ratio, **HbA1c:** Hemoglobin A1c.

# Supplementary Figures


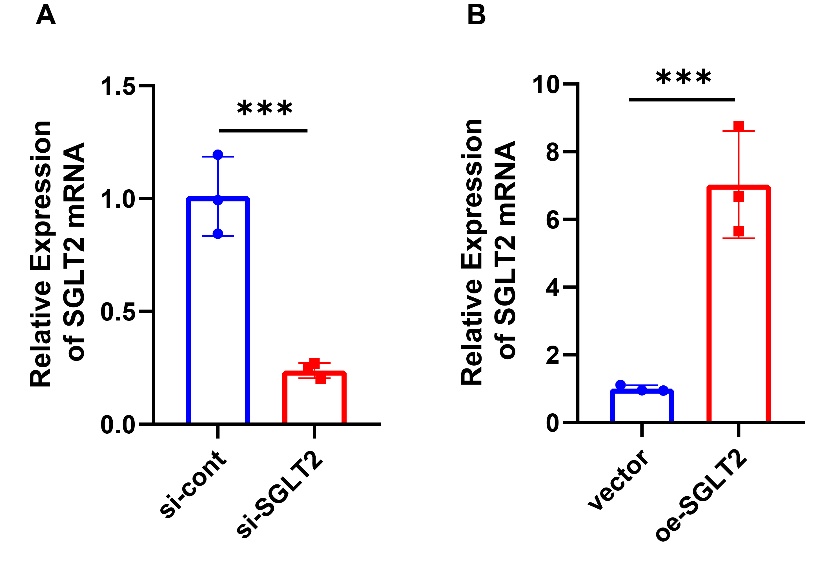


## Supplementary Figure 1. Modulation of SGLT2 expression alters SGLT2 mRNA levels in HK2 cells

(A) Knockdown of SGLT2 using siRNA significantly reduced SGLT2 mRNA expression compared to scrambled siRNA controls. (B) Overexpression of SGLT2 via plasmid transfection increased SGLT2 mRNA levels relative to empty vector controls. Data are presented as mean ± SEM. ***p < 0.001 by Student’s t test.


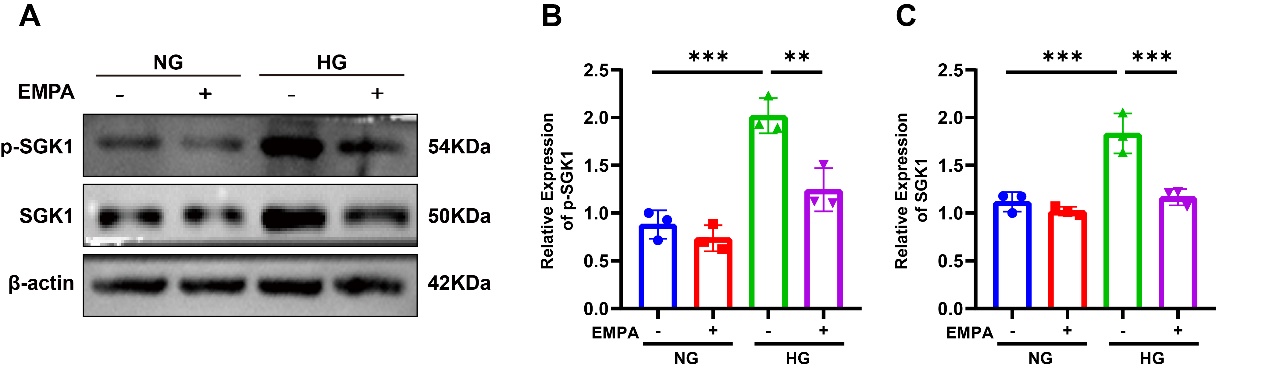


## Supplementary Figure 2. EMPA treatment suppresses HG-induced activation of SGK1 signaling in HK2 cells

(A) Representative western blot images of p-SGK1 and SGK1 expression. (B) Quantification of p-SGK1 levels in HK2 cells. (C) Quantification of total SGK1 levels in HK2 cells. Data are presented as mean ± SEM. **p < 0.01, ***p < 0.001 by one-way ANOVA.
